# The Threshold Effect of Leveraged Trading on the Stock Price Crash Risk: Evidence from China

**DOI:** 10.3390/e22030268

**Published:** 2020-02-26

**Authors:** Zhen Peng, Changsheng Hu

**Affiliations:** 1 School of Business, Hubei University, Wuhan 430062, China; pengzhen@hubu.edu.cn; 2 Economics and Management School, Wuhan University, Wuhan 430072, China

**Keywords:** leveraged trading, stock price crash risk, threshold effect, complexity in stock market

## Abstract

The stock price crash constitutes one part of the complexity in the stock market. We aim to verify the threshold effect of leveraged trading on the stock price crash risk from the perspective of feedback trading. We empirically demonstrate that leveraged trading has a threshold effect on the stock price crash risk on the basis of monthly data on leveraged trading in the Chinese stock market from January 2014 to December 2016. At a low leverage ratio, leveraged trading reduces the stock price crash risk; however, as the leverage ratio increases and exceeds a certain threshold, leveraged trading asymmetrically increases the stock price crash risk. These findings provide new insights in understanding the complexity in the Chinese stock market.

## 1. Introduction

The financial markets are very complex systems; factors of both the internal and the external origin are strongly interrelated by a largely unknown network of connections and feedbacks (positive and negative) [1]. Behavioral finance theory thinks that the abnormal volatility (according to the Efficient Market Hypothesis [2], changes in asset prices should be driven entirely by fundamental information, and the volatility of asset prices should equal the volatility of fundamental factors. Therefore, abnormal volatility is defined as the portion of the volatility of asset prices that exceeds the boundary of the volatility of fundamental factors [3]) is closely related to investors’ feedback trading [3]. In addition, Feedback trading is a very common phenomenon in the financial markets and it can have a significant impact on the complexity of asset prices behavior [4].

The stock price crash constitutes one part of the complexity in the stock market [1]. Controversy surrounding the role of leveraged trading in the stock price crash is ongoing. Some scholars think that leveraged trading will mitigate the impact of arbitrage restrictions and reduce stock price crashes in the stock market [5,6,7]. However, other scholars assert that leveraged trading might expand the influence of private information in the stock market, thus inducing price speculation and increasing stock price crashes [8]. With the establishment of a leveraged trading system in the Chinese stock market in 2010, the Chinese academic community has begun to examine this issue. Some scholars provided evidence that the introduction of short selling can eliminate stock price bubbles and improve market pricing efficiency [9,10]; others reported that leveraged trading will cause the stock price to be overvalued during a bull market and increase the stock price crash risk [11,12].

The severe asymmetry of leveraged trading in the Chinese stock market is an important factor affecting the stock price crash risk. Being constrained by insufficient securities, the high cost of short selling, and the over-optimism of investors during the bull market, the volume of short selling is vastly inferior to the volume of margin trading in the Chinese stock market. The severe asymmetry between margin trading and short selling has weakened the effect of short selling on suppressing price overvaluing [13]. “Strong margin trading and weak short selling” thus characterize leveraged trading. However, leveraged trading plays a role in eliminating arbitrage restrictions and asymmetry also causes more restrictions, thereby forming a complex positive feedback loop, resulting in drastic price volatility, and possibly causing stock prices to crash.

We aim to study the effects of leveraged trading on the stock price crash risk from the perspective of feedback trading. Sentiment feedback trading, changes in asset prices affect investor sentiment, and investor sentiment in turn affects asset prices, is the most representative and well-known in all the types of feedback trading. The premise of sentiment feedback trading is that investor sentiment has a systematic effect on asset prices [14]. Sentiment feedback trading is characterized by the fact that the fundamental factors in the feedback are exogenous variables, and investor sentiment and asset prices have no direct impact on the fundamental factors. Lots of psychological experimental evidence verifies the sentiment feedback trading [15,16].

The motivation of the research is focused on testing whether the leverage ratio has a threshold effect on the stock price crash risk. We empirically demonstrate that leveraged trading has a threshold effect on the stock price crash risk on the basis of monthly data on leveraged trading in the Chinese stock market from January 2014 to December 2016. Specifically, changes in the leverage ratio are closely positively correlated with the stock price crash risk. Under a low leverage ratio, leveraged trading reduces price volatility and the stock price crash risk. However, leveraged trading asymmetrically increases the stock price crash risk, as the leverage ratio increases and exceeds a certain threshold.

This paper adds to the growing literature on the complexity in the stock market by testing whether leveraged trading has a threshold effect on the stock price crash risk, which will provide new insights in understanding the complexity in the Chinese stock market.

A breakdown of the paper is structured, as follows: Section 2 gives the introduction for hypothesis and methodology, Section 3 shows data preprocessing and descriptive statistics, Section 4 gives results presentation and discussion, Section 5 performs some robustness checks, and Section 6 provides the conclusions.

## 2. Hypothesis and Methodology

### 2.1. Hypothesis

The following relationship between the leverage ratio and investors’ feedback trading is proposed on the basis of the sentiment mechanisms of the preferences and beliefs of investors, such as “myopic loss aversion” [17], “mental accounting” [18], “gambling preferences” [19], “realization utility” [20], “herd behavior” [21], “regret aversion bias” [22], and “heterogeneous beliefs” [7,23]: at low leverage ratios, investors tend to exhibit the positive feedback trading behavior of “chasing up and down” to gain higher returns, and at high leverage ratios, investors are more inclined to show the negative feedback trading behavior of “selling high and buying low” to “lock in” profits and avoid losses [24]. Therefore, after the introduction of leveraged trading, the feedback trading pattern of investors is no longer completely random, but somewhat certain. In the model of Hu and Peng [25], it showed that leveraged trading could affect investor sentiment and investors’ feedback trading behavior. When the leverage ratio exceeds a certain threshold, the strong shift of feedback trading from positive to negative will cause stock price crashes. Therefore, we propose the hypothesis, as follows.

**Hypothesis** **1.** *The leverage ratio has a threshold effect on the stock price crash risk.*

### 2.2. Sample Data

July 2014 is generally thought to be the starting point for the abnormal volatility in the Chinese stock market, after which the market entered a bull market and the prices rose rapidly [13]. The prices began to crash in mid-June 2015. Notably, from 12 June to 9 July 2015, the Shanghai Composite Index fell by nearly 35%. At the beginning of January 2016, the Shanghai Composite Index continued to fall. We selected monthly sample data from January 2014 to December 2016 to comprehensively study the mechanism of the leverage ratio on the stock price crash risk under abnormal volatility. The stocks that were eligible for leveraged trading during this period were then selected (the initial sample size is 950 stocks). These data used in this paper were all obtained from the CSMAR (China Stock Market & Accounting Research) database and the Wind database in China.

The sample period of 36 months from January 2014 to December 2016 includes the whole period of abnormal volatility in the recent Chinese stock market, including the phase of stock prices skyrocketing and the phase of stock prices crash. We cannot only study the relationship between leveraged trading and investor sentiment based on this sample, but also the relationship between leveraged trading and abnormal volatility. This is also the main objective of this work. What is more, after 2016, leveraged trading in the Chinese stock market was, to some extent, restricted by the regulations, and was not a completely spontaneous market behavior.

### 2.3. Definition of Core Variables

#### 2.3.1. Leverage Ratio

In this work, market-level data on leveraged trading were used to measure the leverage ratio. The leverage ratio is defined, as shown in Equation (1):

(1) $$tradingleverage_{t}=\frac{financing_{t}-shorting_{t}}{marketvalue_{t}}$$

where *tradingleverage* represents the leverage ratio, *financing* represents the total margin trading balance, *shorting* represents the total short selling balance, and *marketvalue* represents the total market value. The time horizon is monthly. Equation (1) reflects the proportion of the net margin trading in the market at month t to its total tradable market value. The larger the ratio, the higher the proportion of margin trading in the market, which results in a higher leverage ratio.

#### 2.3.2. Stock Price Crash Risk

At present, many types of indicators are used to measure the stock price crash risk. The first is a volatility indicator, which includes volatility, amplitude, and cumulative volatility. The more severe the price volatility, the greater the possibility of a price crash. The second is the distribution of a stock return; if the distribution of stock return is extremely negative (leftward), then the stock return has a large tail risk and it is likely to crash. We measured the stock price crash risk in terms of the skewness of the distribution of a stock return, specifically *DUVOL* (down-to-up volatility) [26,27]. We also used other indicators, such as *NCSKEW* (negative coefficient of skewness) [26,27] and *volatility*, for the empirical test. However, *DUVOL* is consistent with the reality of the stock price crash risks in the Chinese stock market.

We use the model that is shown in Equation (2) to estimate the return of an individual stock after market risk adjustment:

(2) $$r_{i,k}=\alpha +\beta_{1,i}\times r_{m,k-2}+\beta_{2,i}\times r_{m,k-1}+\beta_{3,i}\times r_{m,k}+\beta_{4,i}\times r_{m,k+1}+\beta_{5,i}\times r_{m,k+2}+\varepsilon_{i,k}$$

where $r_{i,k}$ is the return of stock *i* on day *k* and $r_{m,k}$ is the average return of all stocks on day *k* while using the weighted market value. In Equation (2), in addition to adding $r_{m,k}$, we add two lagged terms ($r_{m,k-1}\;$ and $\;r_{m,k-2}$) of market return and two leading terms ($r_{m,k+1}\;$ and $r_{m,k+2}$) of market return to eliminate the effects of asynchronous stock trading. $\varepsilon_{i,k}$ represents the residual after regression. $W_{i,k}=ln\left(1+\varepsilon_{i,k}\right)$ determines the idiosyncratic return of stock *i* on day *k* after market risk adjustment.

Subsequently, used the regression result of Equation (2) to construct *DUVOL*. The daily return data of stock *i* was divided into the up phase (where $W_{i,k}$ was more than $\bar{W}$) and the down phase (where $W_{i,k}$ is less than $\bar{W}$), depending on whether $W_{i,k}$ is greater than the monthly average return $\bar{W}$. Subsequently, we calculated the standard deviation of the returns in these two phases. *DUVOL* is defined, as shown in Equation (3):

(3) $$DUVOL_{i,k}=ln\{\left[\left(n_{u}-1\right)\sum_{down}W_{i,s}^{2}\right]/\left(n_{d}-1\right)\sum_{up}W_{i,s}^{2}]$$

where $\;n_{u}\left(n_{d}\right)\;$ represents the days, in which is $W_{i,k}\;$ more or less than $\bar{W_{i}}$. The larger the value of *DUVOL,* the more serious the negative bias (leftward) of the stock’s idiosyncratic return, which also indicates a higher risk of a stock price crash.

A price crash across the entire market is unavoidable when the risk of a price crash for the main stocks in the market is high. We first calculated the monthly *DUVOL* at the individual stock level and then used the average trend of all stocks’ monthly *DUVOL* values to represent the stock price crash risk at the market level.

### 2.4. Empirical Model

We studied the threshold effect of leverage on the stock price crash risk at the market level. The regression model is defined, as shown in Equation (4):

(4) $$CrashRisk_{t}=\alpha +\beta_{1}\times tradingleverage_{t-1}+\beta_{2}\times dummies\times tradingleverage_{t-1}\;+\varphi \times ControlVariables_{t-1}+\sum^{\;}timevariables+\varepsilon_{t}$$

where the explained variable *CrashRisk* represents the stock price crash risk at the market level (the average value of all stocks’ monthly *DUVOL*). The explanatory variable *tradingleverage* represents the market leverage ratio and *dummies* represents the dummy variables that are used for setting the time threshold and leverage ratio threshold in this work. *dummies* examine whether the impact of the leverage ratio on the stock price crash risk varies with different time thresholds and different leverage ratio thresholds of the Chinese stock market.

In this study, the following control variables were added to the regression model: (1) *retn* represents the stock index return; (2) *lnsize* represents a natural logarithm of the total market value; (3) *turnover* represents the stock turnover rate, which reflects investor sentiment [28]; and, (4) *illiquidity* represents market illiquidity. The ratio of the absolute value of the market return to trading volume is expressed as $illiquidity_{t}=\left|\frac{retn_{t}}{tradingvolume_{t}}\right|$ [29]. Per common practice, we used a first-order lag of all the explanatory variables on the right side of the model [11]. Time dummy variables are also included as controls for the time trend, which could eliminate the risk of spurious regression caused by non-stationarity with time series. Including the robust standard error in the regression controls heteroscedasticity. The time horizon is monthly.

## 3. Data Preprocessing and Descriptive Statistics

### 3.1. Data Preprocessing

We screened the sample stocks and removed the following sample stocks: (1) financial stocks; (2) stocks with abnormal trading status, including ST, * ST, and delisted stocks; (3) stocks in their IPO month; (4) stocks with a long-term suspension (less than 220 trading days in one year); and, (5) stocks that have been disqualified from leveraged trading by the stock exchanges in China. In the Chinese stock market, ST denotes a stock that is specially treated due to bad financial issues; * ST denotes a stock that is specially treated to warn of the risk of the listing being terminated. 815 stocks remained in the sample after the above screening. All continuous variables were subjected to winsorizing according to the 1% and 99% quantiles to control for the influence of extreme values. The missing values in the data were filled while using the linear interpolation method. We then calculated the average of all continuous variables to obtain the average of all sample stocks, which represented the variables at the market level.

### 3.2. Descriptive Statistics

Since the launch of leveraged trading in the Chinese stock market on 31 March 2010, the leveraged trading business has developed rapidly. The balance of margin trading and short selling in the Chinese stock market increased from 12,772 billion RMB in 2010 to 967,961 billion RMB at the end of 2016 and it has continued to grow very rapidly, as shown in Table 1. However, the structural imbalance is serious in China’s margin trading and short selling business. In Table 1, generally speaking, a notable feature is that the size of margin trading accounts for more than 99% of the total balance of leveraged trading, whereas short selling always accounts for less than 1%. Margin trading occupies most of leveraged trading, and the function of short selling is seriously limited. Leveraged trading exhibits are characterized by strong margin trading and weak short selling, which strengthens the arbitrage restrictions in the Chinese stock market. Table 1 shows that the size of margin trading has increased rapidly since 2010; margin trading peaked during the peak of the bull market in 2015 and then began to decline.

Table 2 lists the descriptive statistical results of the variables. The stock price crash risk and leverage ratio show a significantly high volatility at the market level.

### 3.3. Time Trend Fitness of Variables

In Section 3.3, we analyze the time trend fitness of variables, and empirically demonstrate the potential threshold effects of leveraged trading on the stock price crash risk. Although the non-linear effects of the stock price crash risk may vary [30,31]; however, the quadratic effect of leveraged trading on the stock price crash risk has been confirmed by many studies [23,24,32]. Therefore, we compare the linear trend and quadratic trend fitness of variables based on the literatures mentioned above.

**Figure 1:**
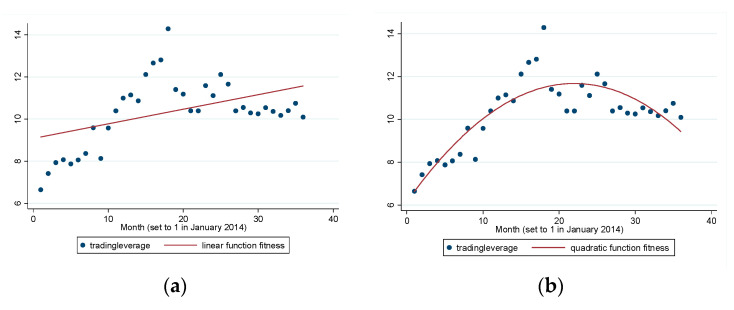
(**a**) Linear function fitness for *tradingleverage* (adj $R^{2}=0.21$) and (**b**) quadratic function fitness for *tradingleverage* (adj $R^{2}=0.69$).

Figure 1a,b show the time trend of the market leverage ratio in terms of linear function fitness and quadratic function fitness. The effect of the quadratic function fitness is better than that of linear fitness because the adjusted $R^{2}$ of the quadratic function fitness is higher than the adjusted $R^{2}$ of linear fitness. *tradingleverage* shows an inverted U-shape distribution over time, increasing during the bull market (July 2014–May 2015), but decreasing during a bear market (July 2014–December 2016). The demarcation point (around June 2014) is in line with the period when the China Securities Regulatory Commission implemented strong measures to deleverage.

**Figure 2:**
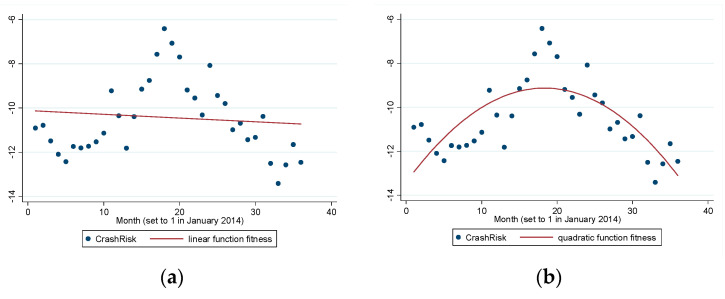
(**a**) Linear function fitness for *CrashRisk* (adj $R^{2}=0.0101$) and (**b**) quadratic function fitness for *CrashRisk* (adj $R^{2}=0.5452$).

Figure 2a,b show the time trend for the market price crash risk. The effect of quadratic function fitness is also better than that of linear fitness, because the adjusted $R^{2}$ of quadratic function fitness is higher than that of linear fitness. *CrashRisk* has an inverted U-shape distribution over time, similar to the distribution of the leverage ratio. *CrashRisk* is relatively low before the start of the bull market, but it increases during the bull market and decreases during the bear market. The demarcation point (around June 2014) is also in line with the date of the Chinese stock market price crash.

### 3.4. Correlation Fitness of Tradingleverage and CrashRisk

**Figure 3:**
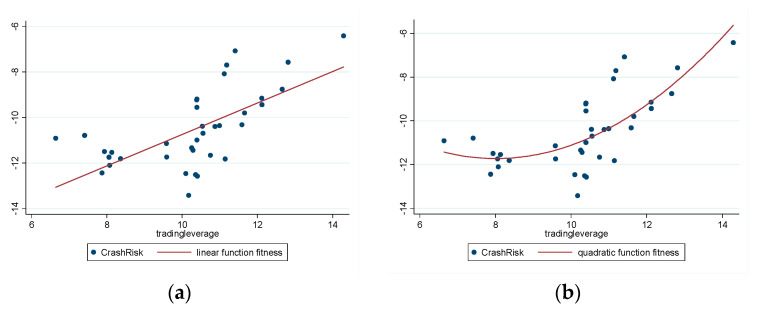
(**a**) Linear function fitness for *tradingleverage* and *CrashRisk* (adj $R^{2}=0.4063$) and (**b**) quadratic function fitness for *tradingleverage* and *CrashRisk* (adj $R^{2}=0.5708$).

Figure 3a,b show the fitness of *tradingleverage* and *CrashRisk*. Here, the effect of the quadratic function fitness is also better than that for linear fitness because the adjusted $R^{2}$ of quadratic function fitness is higher than that of linear fitness.

Figure 3b shows that the leverage ratio has a non-linear effect on the stock price crash risk, and the leverage ratio threshold point is about 10%. When *tradingleverage* is less than 10%, an increase in *tradingleverage* does not significantly increase *CrashRisk* and it might have reduced *CrashRisk*. When *tradingleverage* exceeds 10%, an increase in *tradingleverage* significantly increases *CrashRisk*. When comparing Figure 1b and Figure 3b, the time point corresponding to the leverage ratio threshold is around October 2014. When *tradingleverage* reaches its maximum value (about 14%), *CrashRisk* also reaches its maximum (about −6), as shown in Figure 3b. Comparing Figure 1b and Figure 2b shows that June 2015 has the highest *tradingleverage* and highest *CrashRisk*, which is the date of the Chinese stock market price crash.

**Figure 4:**
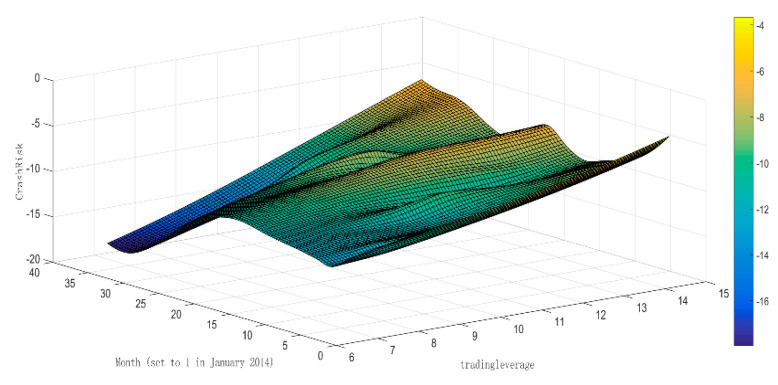
The relationship among *tradingleverage*, *month*, and *CrashRisk.* The lighter the color, the greater the *CrashRisk*.

Figure 4 shows the relationship between *tradingleverage*, *month*, and *CrashRisk* to further depict the relationship of the leverage ratio and the stock price crash risk. The lighter the color, the greater the *CrashRisk*. As per Figure 4, as leverage ratio increases, the color becomes lighter, and the stock price crash risk increases; over time, the color lightens and then turns dark, which means that the stock price crash risk initially increases but decreases after a period of time. Figure 4 confirms the correlation between the leverage ratio and the stock price crash risk.

The time dummy variables are set, as follows: *dummyt1* (take a value of 1 if the time is after September 2014; otherwise, take 0); *dummyt2* (take a value of 1 if the time is after May 2015; otherwise, take 0). A dummy variable of the leverage ratio is then set: *dummylev* (take a value of 1 when the leverage ratio is greater than 10%; otherwise, take 0). The second leverage ratio threshold (potential threshold) could only be artificially set to 14% for June 2015, which is the date of the Chinese stock market price crash, since the maximum leverage ratio in the sample is about 14%.

## 4. Results Presentation and Discussion

We examined the threshold effect of leveraged trading on the stock price crash risk according to Equation (4). Table 3 shows the results.

The endogeneity issue deserves attention in the regression model. It is necessary to control the variables related to *tradingleverage* that also affect *CrashRisk* in the regression model, so as to reduce the impact of the endogeneity issue as much as possible in order to ensure that the thresholds of leveraged trading on the stock price crash risk are unbiased in the empirical results. In the recent studies, the variables related to *tradingleverage* that also affect *CrashRisk* are the following four categories: (1) the stock index return (*retn*) [26,33]; (2) investor sentiment (*turnover*) [24,28]; (3) market liquidity (*illiquidity*) [29,32]; and, (4) the total market value (*lnsize*) [33]. In Table 3, we controlled the above variables, which can effectively reduce the interference of the endogeneity issue on the results.

Table 3 shows the regression result of the leverage ratio to the stock price crash risk. This result is subdivided into four models. Model 1 tested whether a linear relationship existed between the leverage ratio to the stock price crash risk; Model 2–Model 4 further examined whether a threshold effect of the leverage ratio to the stock price crash risk existed. Model 2 tested whether October 2014 was a time threshold and if exceeding this time threshold increased the stock price crash risk. Model 3 tested whether June 2015 was another time threshold and whether exceeding this time threshold caused the stock price to crash. Model 4 tested whether 10% of the leverage ratio was the leverage ratio threshold and whether exceeding this leverage ratio threshold increased the stock price crash risk.

Model 1 shows that *tradingleverage* is significantly positive, and an increase in *tradingleverage* significantly increases *CrashRisk*; *turnover* is also significantly positive, which means that high investor sentiment promotes the stock price crash risk. *illiquidity* is significantly positive, which indicates that a deterioration in market liquidity significantly increases the stock price crash risk, which is consistent with Wei et al. [34].

Model 2 shows that *dummyt1* × *tradingleverage* is significantly positive, and the magnitude of the effect is relatively large, thus further verifying that October 2014 is one of time thresholds. Before October 2014, the increase in the leverage ratio reduces the stock price crash risk and maintains stock market stability during this period. However, after October 2014, the leverage ratio asymmetrically increases the stock price crash risk. A high investor sentiment and deterioration of market liquidity will significantly increase the stock price crash risk.

Model 3 shows that *dummyt2* × *tradingleverage* is significantly positive, and the magnitude of the effect is relatively large, thus further verifying that June 2015 is another time threshold. After June 2015, the increase in the leverage ratio boosts price volatility and eventually asymmetrically increases the stock price crash risk.

Model 4 shows that *dummylev* × *tradingleverage* is significantly positive, and the magnitude of the effect is relatively large, thus indicating that the threshold effect of leveraged trading on the stock price crash risk occurs at around the 10% of leverage ratio. 10% of the leverage ratio is a significant threshold. When the leverage ratio is less than 10%, an increase in the leverage ratio reduces the stock price crash risk and maintains the stock market stability. However, the leverage ratio asymmetrically increases the stock price crash risk when the leverage ratio exceeds 10%. The leverage ratio threshold reveals that leveraged trading has an asymmetric effect on stock market stability. High investor sentiment will significantly increase the stock price crash risk, but the deterioration of market liquidity will significantly decrease the stock price crash risk. Of the leverage ratio, 10%, is also the turning point of feedback trading pattern of investors shift from the positive feedback trading behavior of “chasing up and down” to the negative feedback trading behavior of “selling high and buying low” [24]. The strong shift of feedback trading from positive to negative will indeed cause stock price crashes when the leverage ratio exceeds a certain threshold [25].

We discuss the conclusions with the previous studies. First, the empirical results verify the threshold effect of leveraged trading on the stock price crash risk. We verified that October 2014 and June 2015 are two significant time thresholds, and 10% of the leverage ratio is a significant leverage ratio threshold, which is consistent with the models [7,23,25] and the empirical results [24,34]. The important roles of investor behavior and market liquidity on the stock price crash risk have been confirmed. Second, the conclusions are different from the rational financial theory. The rational financial theory asserts that leveraged trading is a rational behavior of arbitrage, and leveraged trading maintains the stock market as being sustainable [6,7,8]. However, we find that leveraged trading plays a positive role in mitigating arbitrage restrictions and maintaining market stability in the stage with a low leverage ratio, but it has a negative role in exacerbating sentiment feedback trading, and increasing stock price crashes under a high leverage ratio. The effect of leveraged trading on the stock price crash risk is two-edged.

Thus far, the hypothesis is verified.

## 5. Robustness Test

The discussions in this work have limitations. Before examination of the threshold effect, the possible threshold values are pre-judged based on the fitness of the variables’ correlation, which is somewhat subjective. We re-collected the market level data and used the threshold regression model for an in-depth analysis to test the robustness of the results [35].

### 5.1. Methodology

Threshold regression rolls the sample to test all potential thresholds and, therefore, requires a sufficient sample size. We re-collected the daily data of the stock index from 1 January 2014 to 31 December 2016. The threshold effect regression model was constructed, as shown in Equation (5).

(5) $$CrashRisk_{t}=\alpha +\beta_{1}\times tradingleverage_{t-1}\times I\left(q_{i}\leq \gamma \right)\;+\beta_{2}\times tradingleverage_{t-1}\times I\left(q_{i}\geq \gamma \right)\;+\varphi \times ControlVariables_{t-1}+\sum^{\;}timevariables+\varepsilon_{t}$$

where $q_{i}$ represents a series of potential thresholds (including time thresholds and leverage ratio thresholds). $Iq_{i}\leq \gamma$ is an illustrative function, which takes 1 if the expression in the parentheses is true; otherwise, it takes 0. The time horizon is daily.

### 5.2. Threshold Regression Results and Discussion

We used a threshold regression model to re-examine the thresholds (including the time thresholds and leverage ratio thresholds). Table 4 shows the regression results of the time thresholds and Table 5 provides the results of the leverage ratio thresholds.

Table 4 verifies that 20 October 2014 and 28 May 2015 are the two significant time thresholds, which is consistent with the results presented in Section 4. Table 5 also verifies that 10.2% and 13.6% are the two significant leverage ratio thresholds. The magnitudes of the threshold effect are relatively large. When compared with Figure 1a, 10.2% of the leverage ratio corresponds to around 4 September 2014, and 13.6% of the leverage ratio corresponds to around 3 July 2015. These time points are slightly later than the results that are presented in Section 4.

## 6. Conclusions

The stock price crash constitutes one part of the complexity in the stock market, and we studied the effect of leveraged trading on the stock price crash risk from the perspective of feedback trading. The findings that are presented here confirm that leveraged trading has the threshold effects (both in the time dimension and the leverage ratio dimension) on the stock price crash risk. We found that leveraged trading not only plays a positive role in mitigating arbitrage restrictions and maintaining market stability in the stage with a low leverage ratio, but it also has a negative role in exacerbating sentiment feedback trading and increasing stock price crashes under a high leverage ratio. Generally speaking, these findings provide new insights in understanding the complexity in the Chinese stock market.

However, this paper has several limitations that require future study. First, we only consider “the whole period of abnormal volatility”, and this leads to a risk of data snooping. Therefore, high frequency data and more recent data should be applied for further robustness test and sensitivity analysis of the results. Second, conducting country comparisons and analyzing the differences between the stock markets in different countries will make sense. For example, developed financial markets and emerging markets significantly differ in terms of investor structure and transaction institution. By country comparisons, we can analyze the impact of the factors mentioned above on leveraged trading and the stock price crash risk. Third, the method of computational finance can be applied in order to analyze the micro mechanism of leveraged trading affecting investors’ behavior. For example, we can use the method of computational finance to build an artificial stock market where investors use leveraged trading to conduct behavioral gaming, which helps to search for more complex dynamic patterns in the relationship between leveraged trading and the stock price crash risk. Therefore, more investigations should be conducted for enhancing the reliability and applicability of the research results.

## Figures and Tables

**Table 1:** Annual changes in the balance of Chinese leveraged trading.

Year	Total Balance of Leveraged Trading (Billion RMB)	Balance of Margin Trading (Billion RMB)	The Balance of Short Selling (Billion RMB)	Proportion of Margin Trading in Total Leveraged Trading	Proportion of Short-Selling in Total Leveraged Trading
2010	12.7720	12.7610	0.0110	0.999	0.001
2011	38.2070	37.5480	0.0650	0.983	0.002
2012	89.5160	85.6940	3.8210	0.957	0.043
2013	346.5270	343.700	3.0570	0.991	0.009
2014	1025.6560	1017.3730	8.2830	0.992	0.008
2015	1174.2670	1171.3070	2.9600	0.997	0.003
2016	967.9610	963.6710	4.2900	0.996	0.004

Data source: The Wind database in China.

**Table 2:** Descriptive statistics of variables.

Variable	Sample Size	Mean	Standard Deviation	Min	Max
Explained variable: Stock price crash risk					
*CrashRisk*	36	−10.4478	1.6882	−13.4223	−6.4126
Core explanatory variable: Leverage ratio					
*tradingleverage*	36	10.4236	1.5542	6.6421	14.2860
Control variables					
*retn*	36	2.2086	9.5035	−27.7085	19.6621
*lnsize*	36	5.0406	0.2104	4.6640	5.5149
*turnover*	36	84.0669	36.5198	34.1426	162.0876
*illiquidity*	36	0.2606	0.1470	0.1097	0.8251

**Table 3:** Regression results of the leverage ratio to the stock price crash risk.

	Model 1	Model 2	Model 3	Model 4
Variable	*CrashRisk_t_*			
*tradingleverage_t−1_*	0.181 **	−0.112 ***	0.254 ***	−0.0897 ***
	(0.00858)	(0.00655)	(0.00706)	(0.00576)
*dummyt1* × *tradingleverage_t−1_*		0.223 ***		
		(0.00345)		
*dummyt2* × *tradingleverage_t−1_*			0.148 ***	
			(0.00166)	
*dummylev* × *tradingleverage_t−1_*				0.289 ***
				(0.00204)
*retn_t−1_*	−0.0530 ***	−0.0552 ***	−0.0196	−0.0564 ***
	(0.0154)	(0.0152)	(0.0150)	(0.0151)
*turnover_t−1_*	0.0213 ***	0.0200 ***	0.0148 **	0.0194 **
	(0.00704)	(0.00717)	(0.00678)	(0.00739)
*illiquidity_t−1_*	0.196 ***	0.202 ***	0.259 ***	−0.513 ***
	(0.0365)	(0.0340)	(0.0205)	(0.0323)
*lnsize_t−1_*	3.462	3.662	3.237	3.490
	(2.391)	(2.473)	(1.947)	(2.502)
constant	−30.33 ***	−29.44 ***	−29.37 ***	−28.32 **
	(9.528)	(10.09)	(8.157)	(10.60)
Time trend	control	control	control	control
*N*	35	35	35	35
Adj *R^2^*	0.731	0.735	0.779	0.739
F statistic	49.55 ***	47.78 ***	26.54 ***	54.49 ***
Portmanteau (Q) statistic	14.16	15.88	23.91	12.67

Note: The standard error in brackets is a robust standard error. **, and *** denote significance at 10%, 5%, and 1% significance levels, respectively. Adj *R*^2^ measures the goodness of fit of the model. F statistic is used to test significance of the model. The F statistics are significant at the 10% significance level in Model 1 to Model 4. Therefore, we can accept the hypothesis of significance of the model. The Portmanteau (Q) statistic is used to test time series autocorrelation. The Portmanteau (Q) statistics are not significant at the 10% significance level in Model 1 to Model 4. Therefore, we can accept the null hypothesis of no autocorrelation.

**Table 4:** The regression results of the time thresholds.

Variables	Coefficient	Robust Standard Error	Z Statistic	*p* Value	95% Confidence Interval	
**Explained Variable: *CrashRisk***						
*retn_t−1_*	0.023 ***	0.006	4.1	0.000	0.012	0.035
*turnover_t−1_*	0.002 ***	0.0003	6.380	0.000	0.001	0.002
*illiquidity_t−1_*	−0.059	0.084	−0.710	0.479	−0.22	0.105
*lnsize_t−1_*	0.007 **	0.003	2.040	0.041	0.000	0.013
Regime 1	Before 20 October 2014					
*tradingleverage_t−1_*	0.006 ***	0.0008	7.010	0.000	0.004	0.008
constant	−0.066 *	0.037	−1.790	0.074	−0.138	0.006
Regime 2	Between 20 October 2014 and 28 May 2015					
*tradingleverage_t−1_*	0.003 ***	0.001	4.970	0.000	0.018	0.004
constant	−0.040	0.040	−1.010	0.311	−0.118	0.038
Regime 3	After 28 May 2015					
*tradingleverage_t−1_*	0.018 ***	0.001	26.150	0.000	0.017	0.020
constant	−0.089 **	0.039	−2.270	0.023	−0.166	−0.012
time thresholds	(1)	20 October 2014		SSE	0.009	
	(2)	28 May 2015		SSE	0.006	

Note: The standard error in brackets is a robust standard error. *, **, and *** denote significance at 10%, 5%, and 1% significance levels, respectively. SSE represents sum squared residual.

**Table 5:** The regression results of the leverage ratio thresholds.

Variables	Coefficient	Robust Standard Error	Z Statistic	*p* Value	95% Confidence Interval	
**Explained Variable: *CrashRisk***						
*retn_t−1_*	0.003	0.010	0.310	0.76	−0.016	0.022
*turnover_t−1_*	0.002 ***	0.0004	4.820	0.00	0.001	0.002
*illiquidity_t−1_*	0.149	0.123	1.210	0.23	−0.093	0.391
*lnsize_t−1_*	0.041 ***	0.002	17.270	0.00	0.036	0.045
Regime 1	Leverage ratio is below 10.2%					
*tradingleverage_t−1_*	−0.02 ***	0.002	−12.98	0.00	−0.027	−0.020
constant	−0.43 ***	0.026	−16.65	0.00	−0.475	−0.375
Regime 2	Leverage ratio is between 10.2% and 13.6%					
*tradingleverage_t−1_*	0.005 ***	0.0007	6.110	0.00	0.003	0.006
constant	−0.48 ***	0.028	−17.06	0.00	−0.539	−0.428
Regime 3	Leverage ratio is higher than 13.6%					
*tradingleverage_t−1_*	0.010 ***	0.002	4.46	0.00	0.006	0.015
constant	−0.51 ***	0.028	−18.47	0.00	−0.567	−0.458
leverage ratio thresholds	(1)	10.2%		SSE	0.0124	
	(2)	13.6%		SSE	0.0112	

Note: The standard error in brackets is a robust standard error. *, **, and *** denote significance at 10%, 5%, and 1% significance levels, respectively. SSE represents sum squared residual.
